# Assessment of ICD eligibility in non-ischaemic cardiomyopathy patients: a position statement by the Task Force of the Dutch Society of Cardiology

**DOI:** 10.1007/s12471-024-01859-7

**Published:** 2024-04-18

**Authors:** Anne-Lotte C. J. van der Lingen, Tom E. Verstraelen, Lieselot van Erven, Joan G. Meeder, Dominic A. Theuns, Kevin Vernooy, Arthur A. M. Wilde, Alexander H. Maass, Cornelis P. Allaart

**Affiliations:** 1 Department of Cardiology, Amsterdam UMC, Amsterdam Cardiovascular Sciences, Vrije Universiteit Amsterdam, Amsterdam, The Netherlands; 2 Department of Cardiology, Heart Centre, Amsterdam UMC, location AMC, Amsterdam, The Netherlands; 3 Department of Cardiology, Leiden University Medical Centre, Leiden, The Netherlands; 4 Department of Cardiology, VieCuri Medical Centre Noord-Limburg, Venlo, The Netherlands; 5 Department of Cardiology, Erasmus University Medical Centre, Rotterdam, The Netherlands; 6 Department of Cardiology, Cardiovascular Research Institute Maastricht, Maastricht University Medical Centre, Maastricht, The Netherlands; 7 Department of Cardiology, University Medical Centre Groningen, Heart Centre, University of Groningen, Groningen, The Netherlands

**Keywords:** Non-ischaemic cardiomyopathy, Implantable cardioverter defibrillator, Mortality, Sudden cardiac death

## Abstract

International guidelines recommend implantation of an implantable cardioverter-defibrillator (ICD) in non-ischaemic cardiomyopathy (NICM) patients with a left ventricular ejection fraction (LVEF) below 35% despite optimal medical therapy and a life expectancy of more than 1 year with good functional status. We propose refinement of these recommendations in patients with NICM, with careful consideration of additional risk parameters for both arrhythmic and non-arrhythmic death. These additional parameters include late gadolinium enhancement on cardiac magnetic resonance imaging and genetic testing for high-risk genetic variants to further assess arrhythmic risk, and age, comorbidities and sex for assessment of non-arrhythmic mortality risk. Moreover, several risk modifiers should be taken into account, such as concomitant arrhythmias that may affect LVEF (atrial fibrillation, premature ventricular beats) and resynchronisation therapy. Even though currently no valid cut-off values have been established, the proposed approach provides a more careful consideration of risks that may result in withholding ICD implantation in patients with low arrhythmic risk and substantial non-arrhythmic mortality risk.

## Introduction

Heart failure with reduced ejection fraction (HFrEF) carries a significant mortality risk with progressive heart failure and arrhythmic events as predominant causes of death [1]. To prevent arrhythmic death, international guidelines recommend implantable cardioverter defibrillator (ICD) implantation in symptomatic patients with a left ventricular ejection fraction (LVEF) below 35%, despite optimal medical therapy (OMT) and with a life expectancy of more than 1 year [2, 3]. In the HFrEF population suffering from ischaemic cardiomyopathy (ICM), these guidelines are based on randomised trials that have consistently shown a survival benefit of ICD therapy [4–7]. For HFrEF patients with non-ischaemic cardiomyopathy (NICM), however, individual randomised trials have demonstrated varying results, and guideline recommendations are based on meta-analyses [3, 5–8].

Most of these randomised trials were performed over two decades ago. Since then, drug therapies for heart failure have improved and adherence to evidence-based medication has increased, resulting in a substantial decline in the incidence of both all-cause mortality and sudden cardiac death (SCD) [9]. This decrease in SCD reignited the discussion specifically on ICD eligibility for NICM patients and was further fuelled by the results of the contemporary DANISH trial, which showed no reduction in all-cause mortality after ICD implantation in NICM patients with HFrEF [8]. However, the DANISH trial has been criticised for mixing two treatment strategies, as the majority of enrolled patients received cardiac resynchronisation therapy (CRT), thus obscuring the results of ICD-only therapy. Indeed, other non-randomised contemporary ICD studies showed a significant benefit of ICD-only implantation in NICM patients, and similar rates of appropriate device therapy between ICM and NICM patients were observed [10–12].

The past two decades have provided new insights into risk stratification for SCD in the heart failure population. As NICM—by definition—includes all causes of cardiomyopathy other than ischaemic heart disease, each underlying aetiology may carry a different risk. Specific genetic variants carrying an increased arrhythmic risk, as well as more general arrhythmic risk stratifiers including late gadolinium enhancement (LGE) on cardiac magnetic resonance imaging (CMR), and risk modifiers such as CRT have been identified. In addition, new insights have been obtained regarding competing risks (i.e. risk factors increasing the likelihood of non-arrhythmic death) against which ICD implantation will not protect. This obviates the need for a more individualised approach weighing arrhythmic risk, competing risks and possible risk modifiers to assess eligibility for ICD implantation in NICM patients. Based on these new insights, the present article aims to provide a framework to refine selection of NICM patients for ICD implantation eligibility beyond the current guidelines. This review will not discuss the use of prophylactic ICD implantation in patients with hypertrophic cardiomyopathy (HCM) or arrhythmogenic right ventricular cardiomyopathy (ARVC), as eligibility criteria for primary prevention therapy for these two NICM entities are not restricted to a LVEF < 35%. For primary prevention ICD therapy in HCM and ARVC we refer readers to the criteria in the ESC guidelines [3].

## Optimal therapy prior to deciding on ICD implantation

As stated in the guidelines, patients should be on stable OMT, including a beta-blocker, angiotensin-converting enzyme (ACE) inhibitor or angiotensin receptor blocker (ARB) and mineralocorticoid receptor antagonist, before being evaluated for ICD implantation [2, 3]. Currently, however, OMT consists of more components than in the earlier guidelines, as the replacement of ACE inhibitor/ARB by angiotensin-receptor neprilysin inhibitors (ARNIs) and the addition of sodium-glucose cotransporter 2 (SGLT2) inhibitors have been shown to reduce symptoms, hospitalisation as well as mortality, and may lead to an increase in LVEF [9, 13].

In addition to OMT, efforts should be directed towards treatment of underlying causes of NICM, requiring a full diagnostic work-up prior to evaluation for ICD. Specific attention should be paid to the presence of types of supraventricular tachycardia, such as atrial fibrillation and atrial flutter, and frequent premature ventricular beats (PVBs), as these arrhythmias may be both the consequence and cause of NICM, and their ongoing presence may lead to further deterioration of cardiac function. PVBs are often a consequence of the cardiomyopathy. However, when one of these arrhythmias develops prior to or during the NICM disease process, further evaluation is mandatory. Generally, a PVB burden ≥ 10% is considered to be contributing to ventricular dysfunction [14]. LV dysfunction due to either of these arrhythmias may require rigorous attempts to preserve stable sinus rhythm, including antiarrhythmic medication and/or ablation [14, 15].

## Ejection fraction and myocardial fibrosis

Among traditional risk factors, LVEF is the strongest independent predictor of SCD and the key parameter in guiding the decision regarding prophylactic ICD implantation [3]. However, its specificity for predicting SCD is limited, since LVEF is related to SCD as well as to cardiovascular death in general. Sensitivity for the occurrence of SCD is also poor, as approximately 6% of prophylactic ICD patients receive appropriate ICD shocks after 2 years of follow-up [11] whereas, in absolute terms, the majority of SCD events occur in individuals with a preserved LVEF [16].

In NICM patients, an additional risk factor more specific to ventricular arrhythmias may be obtained by CMR with LGE. The presence of myocardial fibrosis provides prognostic information, as it may facilitate re-entry tachyarrhythmia [17]. Approximately 45% of NICM patients have myocardial fibrosis present on LGE-CMR, typically with a septal midwall and/or subepicardial location [18]. Multiple studies have shown that the presence of myocardial fibrosis is associated with SCD and mortality, independent of LVEF [18–21]. Klem et al. showed in 1020 NICM patients with a LVEF < 50% that both myocardial fibrosis and LVEF < 35% were independently associated with mortality. However, myocardial fibrosis was strongly related to arrhythmic events, whereas LVEF was not [19]. These results are in line with a meta-analysis showing that midwall fibrosis was independently associated with ventricular arrhythmias [18]. Interestingly, a study evaluating NICM patients with a LVEF > 40%, therefore not considered eligible for ICD implantation, showed that NICM patients with LGE had a nine-fold higher risk of SCD compared to patients without LGE [20]. This is in line with another study showing that patients with a LVEF > 35% with myocardial fibrosis are more prone to arrhythmic events compared to patients with a LVEF between 21 and 35% without myocardial fibrosis [21]. Whereas in ICM the extent of transmural myocardial fibrosis is inversely correlated with the LV function [22], the correlation between the extent of myocardial fibrosis and LV function in most cases of NICM is less obvious. This might be an explanation for the strong correlation of LV dysfunction with arrhythmic events in ICM patients, whereas this correlation is less clear in NICM. As large and prospective studies strongly suggest myocardial fibrosis as a parameter to identify NICM patients at high risk of SCD, we propose that LGE-CMR be incorporated in the standard work-up for NICM, which is in line with the 2A recommendation of the current ESC guidelines [3]. Moreover, if LGE on CMR is absent and the additional evaluation, including genetic testing and cardiomyopathy substrate, shows a low arrhythmic risk, we propose that conservative treatment or CRT without a defibrillator is preferable. Nevertheless, randomised evidence is needed for further validation.

## Risk associated with genetic background

**Fig. 1:**
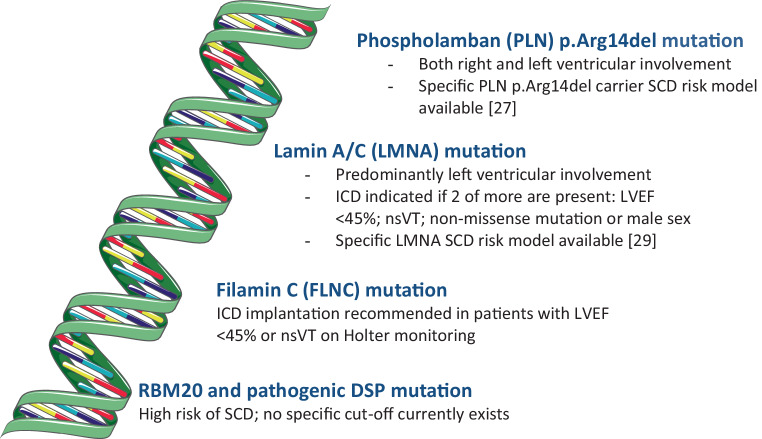
Specific genetic variants and the risk of sudden cardiac death (*SCD*). Currently, most non-ischaemic cardiomyopathy patients with a genetic variant are eligible for implantable cardioverter defibrillator (*ICD*) implantation if their left ventricular ejection fraction (*LVEF*) is ≤ 35%. However, some genetic variants have specific recommendations regarding ICD implantation. (Adapted from SMART—Servier Medical ART, Servier: https://smart.servier.com. *nsVT* non-sustained ventricular tachycardia)

The terms NICM and dilated cardiomyopathy (DCM) are often used interchangeably. However, NICM includes different forms of cardiomyopathies, such as DCM, auto-immune cardiomyopathy, arrhythmia-induced cardiomyopathy etc. DCM is the most common subtype of NICM, and research and recommendations regarding genetic testing have, in contrast to other sections of the article, a specific focus on DCM patients. Approximately 17–25% of DCM cases have a hereditary cause and a pathogenic variant can be found [23]. More than 50 genes are identified to be monogenetically associated with DCM. The most frequent pathogenic variants are in genes coding for titin (TTN), lamin A/C (LMNA) and desmin. In the Netherlands, phospholamban (PLN) mutations are also a common cause because of the p.Arg14del Dutch founder mutation. In a historical cohort, 12% of Dutch DCM cases were found to harbour this PLN mutation [24]. Testing for genetic variants is generally recommended when the prevalence of a detectable pathogenic variant is sufficiently high to justify targeted genetic screening, e.g. in cases of familial DCM, and in patients with onset of disease at a young age without other predisposing factors for DCM [25]. Escobar-Lopez et al. developed a validated screening tool that can predict the probability for a positive genetic test in patients with DCM based on five clinical parameters [26]. It is important to acknowledge that there are gene- or even variant-specific effects. For example, the PLN p.Arg14del variant can lead to both right and left ventricular involvement, while pathogenic variants in LMNA are associated predominantly with left ventricular involvement and conduction abnormalities with or without a skeletal myopathy. Due to these gene- and variant-specific effects, the risk of SCD also varies per genotype. Currently, the LVEF criterion for ICD implantation has been specified for specific genetic backgrounds (Fig. 1). Indeed, for PLN p.Arg14del carriers a specific risk model estimating the risk of lethal arrhythmia was recently developed that can guide eligibility for ICD implantation [27]. In individuals with LMNA, two or more of the following are needed for ICD implantation to be recommended: LVEF < 45%, non-sustained ventricular tachycardia (VT), non-missense mutation or male sex [28, 29]. For filamin C, ICD implantation is recommended for patients with a LVEF < 45% or non-sustained VT on Holter monitoring [30, 31]. DCM patients with an RBM20 (RNA-binding motif protein 20) or pathogenic DSP (desmoplakin) mutation are also considered at high risk of SCD, but no specific cut-off currently exists [23]. In contrast, arrhythmic deaths do occur in TTN mutation carriers, but progressive heart failure predominates. In the future, more specific recommendations are expected for genetic DCM based on additional data on the genotype-phenotype relationship. Importantly, the online calculators for PLN p.Arg14del carriers and LMNA mutation carriers present a 5-year risk of ventricular arrhythmias. Although no specific cut-off values are specified regarding whether or not an ICD should be implanted in these specific patients, the European guideline committee for HCM considered a yearly risk of ≥ 1% of SCD acceptable for prophylactic ICD implantation [32].

## Competing risks: the influence of comorbidities, age and sex

**Fig. 2:**
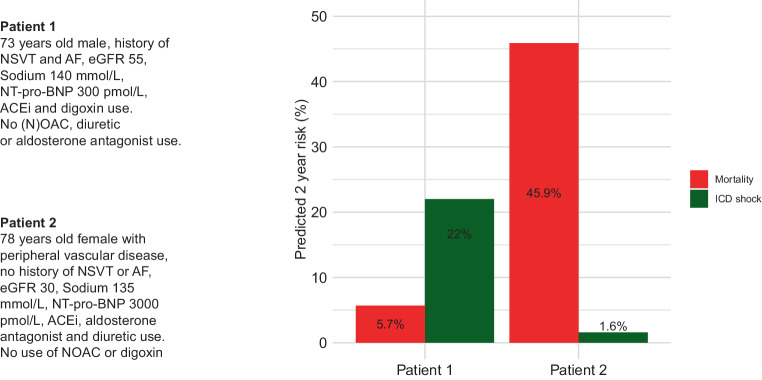
Two sample cases in which the chance of all-cause mortality and implantable cardioverter defibrillator (*ICD*) shock can be used to determine if a patient will benefit from prophylactic ICD implantation. The Do-IT prediction models are used to estimate the individual risk of death and ICD shock. (Adapted from Verstraelen et al. [12] with permission. © 2021; Oxford, Academic. *AF* atrial fibrillation, *ACEi* angiotensin-converting enzyme inhibition, *eGFR* estimated glomerular filtration rate, *(N)OAC* (novel) oral anticoagulant drugs, *nsVT* non-sustained ventricular tachycardia, *NT-pro-BNP* N-terminal pro-B-type natriuretic peptide)

Efficacy of ICD implantation in patients at risk of arrhythmic death may be significantly modified by the risk of non-arrhythmic death. An ICD shock may prevent SCD; however, it does not necessarily prolong life if the chance of non-arrhythmic death in the near future is high. The importance of competing risks has been historically acknowledged in guidelines by stating that life expectancy should be more than 1 year. Presently, however, several validated models including demographic data (e.g. age and sex), as well as comorbidities and other risk markers (e.g. diabetes mellitus, renal failure, chronic obstructive pulmonary disease, N‑terminal pro-B-type natriuretic peptide level), are available for risk assessment in primary prevention ICD implantation and CRT implantation [12, 33]. For example, in the Dutch Do-IT study a validated online tool was developed for calculation of the risks of all-cause mortality and appropriate ICD shock [12]. Importantly, this study shows that risk stratification regarding appropriate device therapy remains challenging compared to predicting mortality, with poor performance for the ICD shock model (C statistic = 0.60) and good performance of the mortality model (C statistic = 0.74). Figure 2 shows two example cases in which the risk of appropriate ICD shock and all-cause mortality is predicted based on clinical parameters. Obviously, increasing age strongly affects mortality risk. A post hoc analysis of the DANISH trial showed that the rate of non-arrhythmic death doubles in NICM patients above the age of 70 years [34]. In line with the DANISH trial, several other studies have shown a decreased benefit of ICD implantation with increasing age [11, 33]. As a consequence, age should be incorporated in clinical decision making for ICD implantation.

Along with age, comorbidities also strongly affect outcome. Almost 60% of HF patients have five or more chronic comorbidities, and this affects their functional status and mortality [35–37]. Studies have shown that, while in elderly patients with multiple comorbidities the benefit of ICD therapy is less clear [37–39], in elderly patients at high risk of SCD with a low burden of comorbidities ICD implantation may increase survival [39]. Importantly, the most recent cardiac pacing guidelines propose CRT‑D implantation based on individual risk assessment rather than a LVEF cut-off alone [40].

In addition to age and comorbidities, sex also stratifies risk. Male sex is associated with a higher all-cause mortality and higher incidence of SCD in ICM and NICM patients [6, 33, 41–43]. A meta-analysis of the pivotal randomised ICD trials including ICM and NICM patients showed that prophylactic ICD implantation is associated with a 25% reduction in mortality in men, whereas ICD implantation in women was not associated with a survival benefit [41]. This may partly be attributable to sex hormones affecting susceptibility for arrhythmia and a lower baseline risk of death in women [42, 44]. However, women have been underrepresented in randomised trials and differences in underlying causes of cardiomyopathy may also partly explain differences in outcome. In addition, female sex is associated with a greater response to CRT compared to males, resulting in improved survival rates and a lower incidence of SCD; yet further analysis suggests that this outcome may be attributable to differences in underlying cardiomyopathy and/or heart size rather than sex per se [45, 46]. As a consequence, incorporation of sex in risk assessment for SCD is less straightforward.

**Fig. 3:**
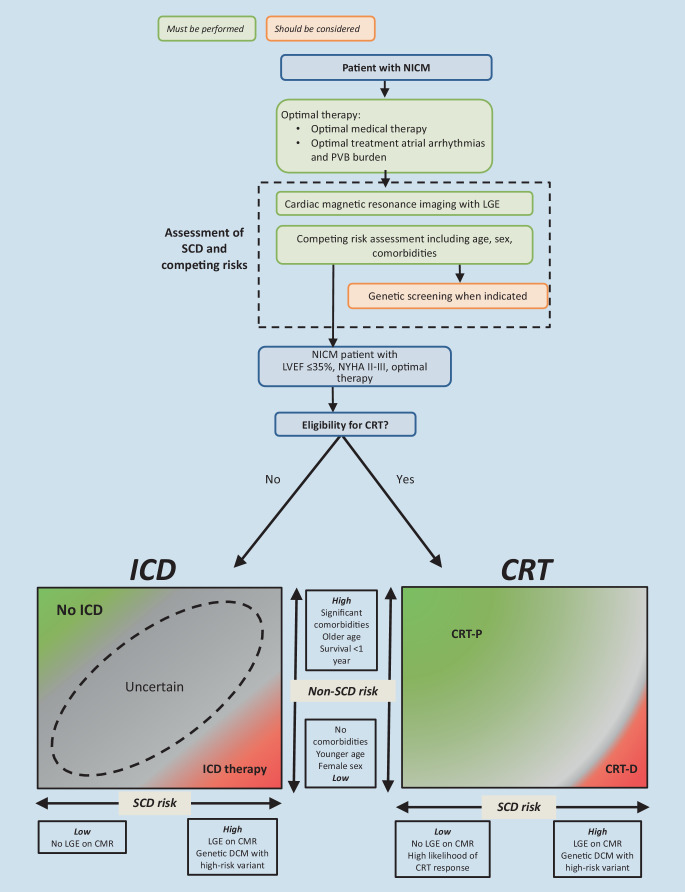
Suggested diagnostic work-up and optimisation of care for non-ischaemic cardiomyopathy (*NICM*) patients. See Fig. 1 and the guidelines for specific recommendations regarding implantable cardioverter defibrillator (*ICD*) recommendations for dilated cardiomyopathy (*DCM*) patients with specific genetic variants. (*CMR* cardiac magnetic resonance, *CRT* cardiac resynchronisation therapy, *LGE* late gadolinium enhancement, *LVEF* left ventricular ejection fraction, *NYHA* New York Heart Association, *PVB* premature ventricular beat, *SCD* sudden cardiac death)

Nevertheless, physicians should estimate risks of both non-arrhythmic and arrhythmic death for each individual patient in order to assess eligibility for ICD implantation. For example, an elderly male NICM patient with several comorbidities, no significant LGE on CMR and no high-risk genetic substrate has a low arrhythmia risk and a substantial risk of non-arrhythmic mortality, and ICD implantation will not significantly improve life expectancy. In contrast, a young patient without comorbidities and with LGE on CMR has a substantial arrhythmic risk and relatively low mortality risk, and may therefore benefit from ICD implantation. Figure 3 depicts the interaction between the risk of arrhythmic death and non-arrhythmic death and can provide insight into the competing risks on a patient level.

## Cardiac resynchronisation therapy

Approximately 35–50% of the NICM patient population eligible for ICD implantation have a ventricular conduction delay amenable to CRT [8, 12]. CRT may result in significant reverse remodelling and improvement of LVEF, and may consequently modify the mortality risk, including the risk of SCD [47]. As a consequence, the need for addition of a defibrillator to CRT therapy has been a subject of debate for a long time. No randomised controlled trials have been performed yet to directly compare the benefit of CRT with a defibrillator function (CRT-D) versus CRT with a pacemaker function (CRT-P) in NICM. However, two meta-analyses using subgroups of randomised and non-randomised studies have become available. These studies show that addition of a defibrillator to CRT therapy was not significantly associated with an overall decrease in all-cause mortality [48, 49]. In contrast, a recent subanalysis of the COMPANION study showed a significantly lower all-cause mortality in NICM patients receiving a CRT‑D compared to those receiving a CRT‑P [50]. It is noteworthy that only patients in New York Heart Association class III–IV were included in this study, with less than 70% of patients receiving ACE inhibitor and beta-blocker treatment, and that this post hoc analysis was relatively underpowered. In general, these findings support the opinion that in a substantial proportion of NICM patients eligible for CRT, a CRT‑P should be preferred over a CRT‑D. Specifically in patients with a high likelihood of CRT-induced reverse remodelling combined with a low estimated arrhythmic risk, such as older patients with a high comorbidity burden, CRT‑P should be considered [51]. The amount of reverse remodelling can be estimated based on patient characteristics and predicted using an easily available effect calculator [52], whereas the risk of SCD can be assessed using previously discussed predictors, including LGE-CMR. The clinical work-up prior to device implantation should not differ between CRT and ICD NICM patients, as LGE-CMR and genetic screening could provide guidance in deciding between CRT‑D and CRT‑P.

## Recommendations

There has been controversy over the usefulness and necessity of primary prevention ICD implantation in HFrEF patients with NICM. Guidelines recommend a decision based on risk assessment using LVEF, OMT and a life expectancy of more than 1 year. In recent decades, however, both the risk of SCD and life expectancy have been modified significantly by improvements in medical therapy. In addition, advances have been made in estimation of arrhythmic as well as non-arrhythmic risks. We therefore propose additional risk assessment refining current guidelines, carefully weighing arrhythmic risk versus non-arrhythmic risks of death for each individual patient (see Infobox 1). Figure 3 shows the proposed approach.

### Infobox 1 Recommendations for further risk assessment

1. Verification of optimal therapy consisting of OMT (including ARNI and/or a SGLT2 inhibitor if possible) as well as optimisation of concomitant arrhythmias that might affect cardiac function.
2. Assessment of arrhythmic risk using LGE-CMR and genetic testing when appropriate.
3. Assessment of non-arrhythmic risk using age, comorbidities and sex.
4. Evaluation of possible risk modifiers, in particular CRT eligibility and its estimated effect size. In a substantial proportion of patients eligible for CRT, a CRT‑P should be preferred over CRT-D: specifically in patients with a high likelihood of CRT-induced reverse remodelling and a low arrhythmic risk.

## Conclusion

This article aims to present a strategy beyond current guidelines for selection of NICM patients eligible for ICD implantation. As randomised evidence is limited, proposed strategies are largely based on observational studies and post hoc analyses. Although we feel that this provides a sufficient basis for the proposed approach, further studies—preferably randomised clinical trials or dedicated registries—are needed particularly for the patient group with intermediate risks.
